# Uncovering sympathetic nervous system dysfunction in disorders of consciousness via heart rate variability during head‐up tilt test

**DOI:** 10.14814/phy2.16000

**Published:** 2024-04-07

**Authors:** Weiqiang Cai, Xu Han, Xinwei Tang, Zuojun Cao, Zi Yu, Zuowen Sun, Junfa Wu, Yi Wu, Hongyu Xie

**Affiliations:** ^1^ Department of Rehabilitation Medicine, Huashan Hospital Fudan University Shanghai China

**Keywords:** disorders of consciousness, head‐up tilt test, heart rate variability, sympathetic nervous system dysfunction

## Abstract

Few standardized tools are available for evaluation of disorders of consciousness (DOC). The potential of heart rate variability (HRV) during head‐up tilt (HUT) test was investigated as a complementary evaluation tool. Twenty‐one DOC patients and 21 healthy participants were enrolled in this study comparing clinical characteristics and HRV time‐ and frequency‐domain outcomes and temporal changes during HUT test. During the 1st–5th min of the HUT, DOC group showed a significant increase and decrease in log low frequency (LF) (*p* = 0.045) and log normalized high frequency (nHF) (*p* = 0.02), respectively, compared to the supine position and had lower log normalized LF (nLF) (*p* = 0.004) and log ratio of low‐to‐high frequency (LF/HF) (*p* = 0.001) compared to healthy controls. As the HUT continued from the 6th to the 20th min, DOC group exhibited a significant increase in log LF/HF (16th–20th min) (*p* < 0.05), along with a decrease in log nHF (6th–10th and 16th–20th min) (*p* < 0.05) and maintained lower log LF, log nLF, and log LF/HF than controls (*p* < 0.05). 1st–10th min after returning to the supine position, DOC group demonstrated a significant decrease in log nHF (*p* < 0.01) and increases in log LF/HF (*p* < 0.01) and had lower log LF (*p* < 0.01) and log nLF (*p* < 0.05) compared to controls. In contrast, the control group exhibited a significant decrease in log nHF (*p* < 0.05) and increase in log LF/HF (*p* < 0.05) throughout the entire HUT test. Notably, no significant differences were observed when comparing time‐domain outcomes reflecting parasympathetic nervous system between the two groups. HRV during HUT test indicated a delayed and attenuated autonomic response, particularly in the sympathetic nervous system, in DOC patients compared with healthy individuals.

## 1 BACKGROUND

Disorders of consciousness (DOC) describe functional impairment lasting for more than 28 days, commonly caused by traumatic brain injury (TBI), cerebral hemorrhage (CH), or hypoxic–ischemic encephalopathy (HIE) (Edlow et al., 2021). DOC can be classified into three categories, according to the level of arousal and consciousness: coma (eyes closed and unable to be woken), unresponsive wakefulness syndrome (UWS)/vegetative state (VS, awake but unable to exhibit any conscious response) and minimally conscious state (MCS, minimal consciousness but clear signs of awareness) (Giacino et al., 2018). Epidemiological data from the United States gives an annual incidence of VS of 4200 cases, with prevalence ranging from 5000 to 42,000, and of MCS from 112,000 to 280,000. The global prevalence of UWS/VS is estimated at 0.2–6.1 cases per 100,000 population (Pistarini & Maggioni, 2021). Subjective and objective evaluation tools, such as the Coma Recovery Scale‐Revised (CRS‐R), electroencephalograms (EEGs) and functional magnetic resonance imaging (fMRI), are available but there is no gold standard tool for evaluating and stratifying DOC (Giacino et al., 2018). Available assessment tools have limitations in clinical practice with, for example, a single CRS‐R evaluation taking 30–40 min and producing discrepant results due to evaluator bias (Edlow et al., 2021). EEGs and fMRIs have demonstrated utility for DOC evaluation and stratification (Edlow et al., 2017; Gosseries et al., 2011) but expensive equipment, complex operational procedures, and challenging data processing have limited their use (Crosson et al., 2010; Puce & Hämäläinen, 2017). Moreover, Patients with DOC resulting from severe TBI often have metal plates due to skull cranioplasty which are not compatible with MRI (Sammet, 2016) and reduced control over body movements results in head movement during scanning, reducing image quality, and diagnostic accuracy. Alternative subjective methods which do not require direct examination through the skull would complement and enhance DOC evaluation.

Heart rate variability (HRV) assessment, at rest or after stimulation, is simple, inexpensive, and noninvasive and reflects the regulation of the autonomic nervous system (ANS), including sympathetic nervous system (SNS) and parasympathetic nervous system (PNS) (Baker et al., 2018). Rapid fluctuations may be detected due to dynamic changes, offering a superior temporal resolution compared to electrophysiology or neuroimaging (Riganello, Chatelle, Schnakers et al., 2019; Riganello, Larroque, Perri et al., 2019). Wijnen et al. have used HRV analysis to demonstrate a positive correlation between consciousness level and ANS regulation in DOC patients (Wijnen et al., 2006) and clinical studies have shown the potential for DOC evaluation and stratification (Liuzzi et al., 2023; Riganello et al., 2018; Riganello, Chatelle, Schnakers et al., 2019). HRV measurements for DOC usually require patients to be in a static supine position which may not fully reflect the dynamic regulation of the ANS. HRV during head‐up tilt (HUT) test reflects changes in sympathetic and parasympathetic excitability by comparing HRV before, during and after positional transition and have been used to diagnose postural tachycardia syndrome and cardioinhibitory vasovagal syncope (Cheshire Jr. & Goldstein, 2019; Miranda & Silva, 2016; Orjatsalo et al., 2020). The potential for DOC evaluation with the possibility of more dynamic and accurate assessment of ANS regulation remains to be explored.

The objective of the study was to examine if a consistent pattern of changes in HRV outcomes existed between DOC patients and healthy participants during the HUT test. Furthermore, the study sought to evaluate whether significant differences in HRV outcomes could be observed between DOC patients and healthy participants, thereby investigating potential variations in autonomic response capacity between these two groups. The formulated hypotheses were as follows: firstly, that the pattern of changes in HRV outcomes during the HUT test would be similar between DOC patients and healthy participants; secondly, that there would be differences in autonomic response capacity between DOC patients and healthy participants, with the latter demonstrating significantly stronger autonomic response capabilities compared to DOC patients.

## 2 MATERIALS AND METHODS

### 2.1 Participants

Patients with DOC who underwent the HUT test at the Department of Rehabilitation Medicine, Huashan Hospital between April 2023 and September 2023 were enrolled. Inclusion criteria were as follows: (a) male or female patients aged 15–80, (b) a diagnosis of DOC, including VS/UWS or MCS, by CRS‐R assessment, (c) ≥28 days since the onset of DOC and (d) DOC of known cause, such as TBI, CH, or HIE. Exclusion criteria were as follows: (a) patients with severe cardiovascular, digestive, kidney, blood, endocrine, respiratory diseases, immunodeficiency, tumors, or other serious diseases and (b) patients who had undergone major surgery within the previous 3 months or received experimental drug/device treatment with unclear effect/safety within the previous 1 month. A group of healthy, age‐ and sex‐matched volunteers who had no habit of smoking and no known previous disease was also enrolled. Written informed consent was given by all healthy participants and by patients' legal representatives prior to the experiment. This study was conducted in accordance with the latest version of the Helsinki Declaration (World Medical Association, 2013) and the protocol was approved by the Ethics Committee of Huashan Hospital (2023 ‐ 469**)** and registered at Chinese Clinical Trial Registry (ChiCTR230007371).

### 2.2 HUT test

**FIGURE 1:**
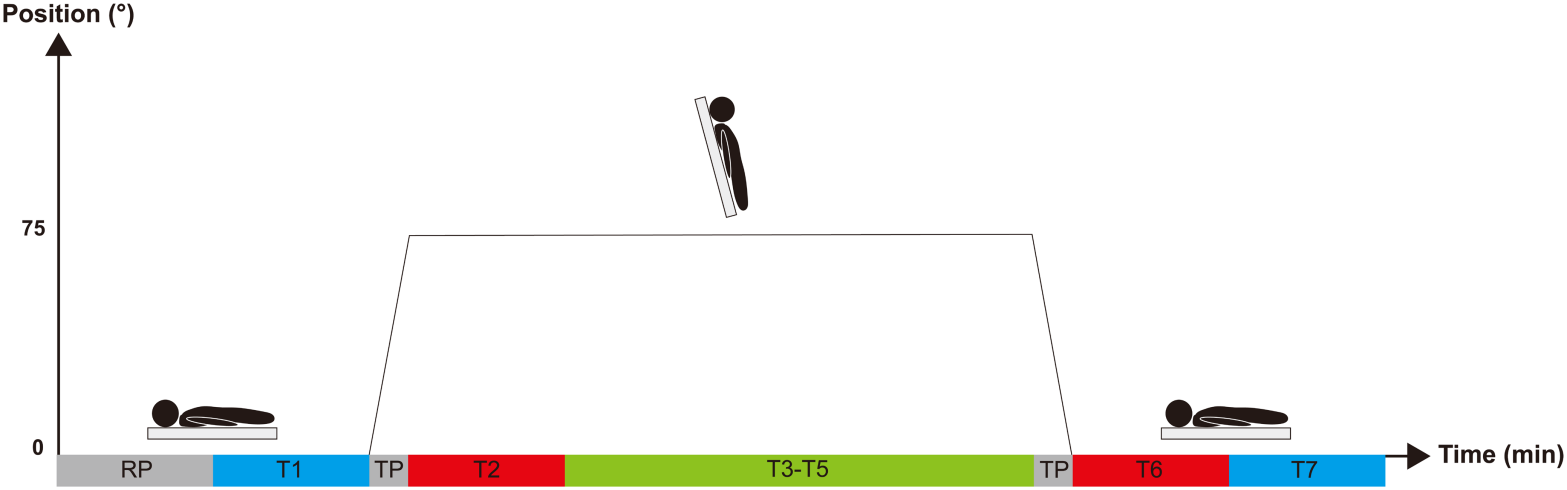
HUT test. HUT: head‐up tilt; RP: 5‐min resting period; T1: 5‐min heart rate variability (HRV) data collection period (baseline supine position); TP: 30 s transitional period (from 0° to 75°; 75° to 0°); T2: first 5‐min HRV data collection period (75° tilted position); T3‐T5: second to fourth 5‐min HRV data periods (75° tilted position); T6: first 5‐min HRV data collection period (supine position after tilt); T7: second 5‐min HRV data collection period (supine position after tilt).

Subjects were instructed to abstain from cigarettes and alcohol for 1 month, avoid caffeine‐containing drinks (such as coffee, tea, and energy drinks) and strenuous exercise for 2 days prior to the HUT test (Minami et al., 1999, Aguilera et al., 1999, Grant et al., 2023, Stanley et al., 2013). The subject lay on the tilt table and three ECG electrodes were applied to the chest and connected to an ECG monitor (mECG‐101, Welld, SZ, China) to record heart rate at a sampling rate of 512 Hz. The HUT test consisted of three phases. Phase one lasted 10.5 min: 5‐min resting period in a supine position, followed by 5‐min HRV data collection (T1) in the same position and 30 s posture transition as the table slowly inclined from 0° to 75° at a constant speed. Phase two lasted 20 min: 5 min HRV data collection (T2) at an angle of 75° immediately following posture transition and three 5‐min data collection stages (T3–T5) at the same inclination. Phase three lasted 10.5 min: 30 s posture transition as the table slowly returned from 75° to 0° at a constant speed and two 5‐min data collection stages (T6 and T7) in a supine position (Figure 1) (Crnošija et al., 2016). All tests took place between 2 pm and 4 pm.

### 2.3 Statistical analysis

Demographic and clinical characteristics of sex, age, CRS‐R score and diagnosis, causes of DOC, days since onset and number of patients who have undergone cranioplasty were collected. HRV data were analyzed by ECG Browser software (Hongkang, SH, China) using time‐ and frequency‐domain methods. Time‐domain outcomes included standard deviation of normal‐to‐normal intervals (SDNN), root mean square of successive differences (RMSSD), and proportion of the number of times successive heartbeat intervals exceeded 50 ms divided by the total number of NN intervals (pNN50). Frequency‐domain outcomes included low frequency (LF, 0.04–0.15 Hz), normalized low frequency (nLF), high frequency (HF, 0.15–0.4 Hz), normalized high‐frequency (nHF), and ratio of low to high frequency (LF/HF) (Shaffer & Ginsberg, 2017). ANS regulation was assessed by SDNN. RMSSD, pNN50, HF, and nHF were used to assess the functional status of the PNS. LF and nLF reflected SNS excitability and the LF/HF ratio indicated the balance between the SNS and PNS.

Data normality was tested with Shapiro–Wilk test, histograms, and quantile‐quantile (Q‐Q) plots. Log_10_ transformation was applied to nonnormal data. Mann–Whitney and Pearson's chi‐squared tests were used to evaluate intergroup differences in age and sex, respectively. A two‐way repeated measures ANOVA was conducted to examine the effect of HUT test on HRV outcomes in DOC patients and healthy participants. It was applied with HRV outcomes under different positions as the dependent variable, and group, position, and the group multiplied by position interaction as independent variables. The group‐by‐position interaction effects and main effects of group and position were tested. Next, between‐group differences at each position and within‐group differences between T1 and other positions were tested by independent and dependent *t*‐test, respectively. SPSS 26.0 software (Armonk, NY, United States) was used for statistical analyses and a *p*‐value <0.05 was considered statistically significant. Effect sizes were computed by using partial eta‐squared (*η*_p_^2^) for two‐way repeated measures ANOVA and Cohen's d for *t*‐test. A *η*_p_^2^ of 0.01 represents a small effect, 0.06 a moderate effect and 0.14 a large effect. A Cohen's d of 0.2 represents a small effect, 0.5 a moderate effect, and 0.8 a large effect (Field, 2005).

## 3 RESULTS

### 3.1 Clinical characteristics

**FIGURE 2:**
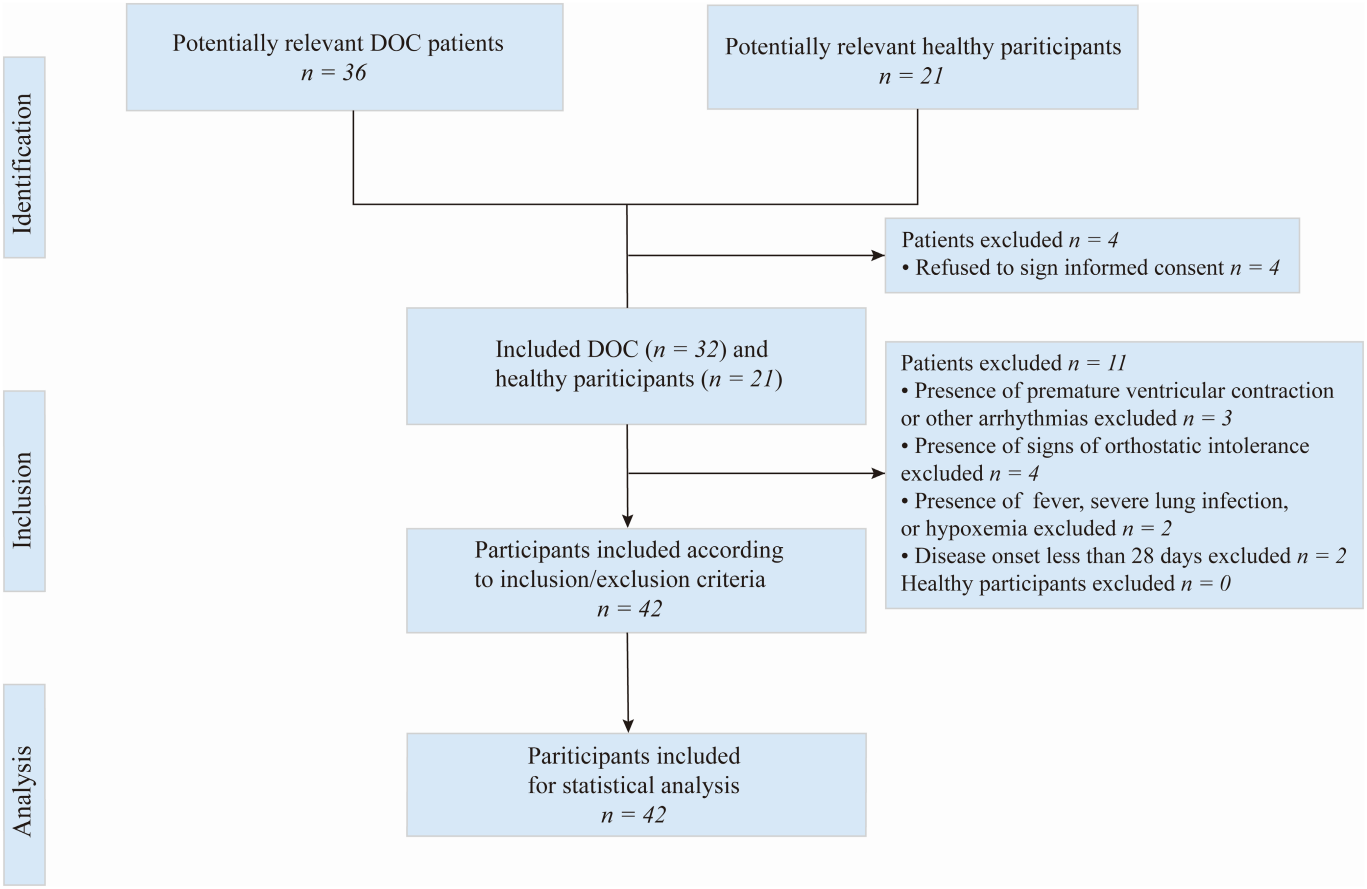
Flowchart of participant selection. DOC, disorders of consciousness.

Forty‐two participants fulfilled the selection criteria, 21 DOC patients, and 21 healthy participants, and 15 DOC patients were excluded (Figure 2). No significant differences in age or sex were found between the two groups (Table 1). DOC patients were divided into three subtypes by CRS‐R score: VS (*n* = 5), MCS− (*n* = 11), and MCS+ (*n* = 5), and 17 patients (80.95%) had undergone cranioplasty (Table 1). DOC etiologies included TBI, CH, and HIE. Mean number of days since DOC onset was 506.14 (SD: 277.48) (Table 1).

**TABLE 1:** Clinical characteristics of DOC patients and healthy controls.

	DOC patients (*n* = 21)		Healthy controls (*n* = 21)	*p*‐Value
Age (years)^a^	47 (34–61.5)		28 (23–55.5)	0.13
Female/male	9:12		10:11	0.76
CRS‐R score^b^ and diagnosis	5.2 (1.48)	VS *n* = 5	/	
	8.82 (2.68)	MCS− *n* = 11		
	16.6 (1.95)	MCS+ *n* = 5		
Etiology	TBI	*n* = 10		
	CH	*n* = 10		
	HIE	*n* = 1		
Days since onset^b^	506.14 (277.48)			
Cranioplasty	Yes	*n* = 17 (80.95%)		
	No	*n* = 4 (19.05%)		

Abbreviations: CH, cerebral hemorrhage; CRS‐R, coma recovery scale‐revised; DOC, disorders of consciousness; HIE, hypoxic–ischemic encephalopathy; MCS, minimally conscious state; TBI, traumatic brain injury; VS, vegetative state.

^a^ Values are medians with interquartile range.

^b^ Values are means with standard deviation.

### 3.2 Two‐way repeated measures ANOVA for time‐ and frequency‐domain outcomes

All the data were normally distributed for each group after log_10_ transformation, as assessed by Shapiro–Wilk test, histogram, and Q‐Q plot, respectively. The assumptions of sphericity were violated in all outcomes, as assessed by Mauchly's Test of Sphericity (*p* < 0.05). Therefore, a Greenhouse–Geisser correction was applied.

There was a statistically significant interaction effect between group and position only on log pNN50 (*F* = 2.75, *p* = 0.03, *η*_p_^2^ = 0.06). Main effects of position were significant on both time‐ and frequency‐domain outcomes (*p* < 0.001, *η*_p_^2^ > 0.06). Main effects of group have significant effects only on log LF (*F* = 13.49, *p* < 0.001, *η*_p_^2^ = 0.25), log nLF (*F* = 25.43, *p* < 0.001, *η*_p_^2^ = 0.39), and log LF/HF (*F* = 7.69, *p* = 0.008, *η*_p_^2^ = 0.16) (Tables S1 and S2).

### 3.3 Temporal changes of time‐ and frequency‐domain outcomes during HUT test within DOC patients and controls

**FIGURE 3:**
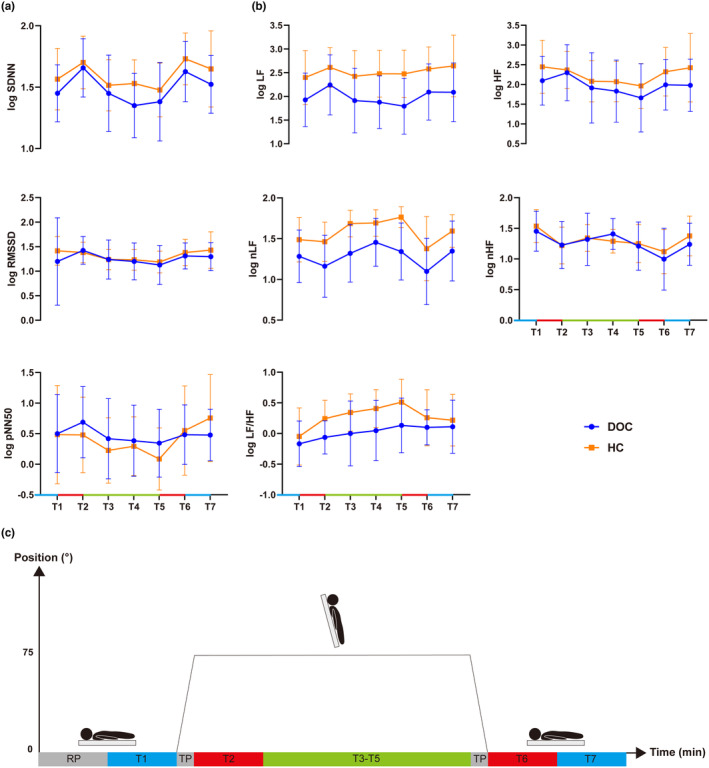
(a, b) Temporal changes in HRV time‐ and frequency‐domain outcomes (normalized by log_10_ transformation) during HUT test for DOC patients and healthy controls. Upper and lower lines are error bars. Dots and squares are means. HRV, heart rate variability; HUT, head‐up tilt; DOC, disorders of consciousness; HC, healthy control; SDNN, standard deviation of normal‐to‐normal intervals; RMSSD, root mean square of successive differences; pNN50: proportion of the number of times successive heartbeat intervals exceeded 50 ms divided by the total number of NN intervals; LF: low frequency; nLF: normalized low frequency; HF: high frequency; nHF: normalized high frequency; LF/HF: low to high‐frequency ratio. (c) illustration of HUT test. RP: 5‐min resting period; T1: 5‐min HRV data collection period (baseline supine position); TP: 30s transitional period (from 0° to 75°; 75° to 0°); T2: first 5‐min HRV data collection period (75° tilted position); T3‐T5: second to fourth 5‐min HRV data periods (75° tilted position); T6: first 5‐min HRV data collection period (supine position after tilt); T7: second 5‐min HRV data collection period (supine position after tilt).

Firstly, temporal changes of time‐domain outcomes, including log SDNN, log RMSSD, and log pNN50, were investigated for each group. An increase in log SDNN for both DOC (*t* = −3.3, *p* = 0.004, Cohen's d = 0.29) and control groups (*t* = −3.11, *p* = 0.005, Cohen's d = 0.2) was seen during T2 (first 5 min at 75° after posture change from supine to 75°) compared to T1 (supine baseline phase) but no significant change in log RMSSD or log pNN50 relative to T1. During T3‐T5 (second to fourth 5‐min intervals at 75° after posture change from supine to 75°), log SDNN showed no significant change in either group compared to T1. RMSSD significantly decreased in the control group (*p* < 0.01) but did not reach significance in the DOC group relative to T1 (Figure 3a,c and Table 2). Log pNN50 showed significant decrease during T5 in the control group (*t* = 2.7, *p* = 0.01, Cohen's d = 0.68), while did not reach significance in the DOC group during T3‐T5 relative to T1.

**TABLE 2:** HRV time‐domain outcomes in HUT test.

	DOC patients (*n* = 21)	Healthy controls (*n* = 21)	Intergroup *p*‐Value	Inter‐group effect size	Within‐group effect size	
					DOC	Healthy control
log SDNN^a^						
T1	1.45 (0.23)	1.57 (0.25)	0.13	0.24	/	
T2	1.66 (0.24)**	1.7 (0.21)**	0.53	0.23	0.29	0.2
T3	1.45 (0.31)	1.52 (0.21)	0.43	0.26	0.35	0.13
T4	1.35 (0.26)	1.53 (0.19)	0.02	0.23	0.27	0.18
T5	1.38 (0.32)	1.48 (0.22)	0.26	0.27	0.29	0.22
T6	1.63 (0.25)*	1.73 (0.21)**	0.15	0.23	0.29	0.19
T7	1.52 (0.24)	1.65 (0.31)	0.15	0.28	0.25	0.19
log RMSSD^a^						
T1	1.37 (0.31)	1.41 (0.29)	0.62	0.3	/	
T2	1.42 (0.28)	1.38 (0.21)	0.58	0.25	0.89	0.24
T3	1.24 (0.4)	1.24 (0.21)**	>0.999	0.32	0.94	0.22
T4	1.2 (0.37)	1.23 (0.21)***	0.73	0.3	0.92	0.19
T5	1.13 (0.4)	1.19 (0.22)***	0.54	0.32	0.96	0.25
T6	1.31 (0.27)	1.38 (0.27)	0.39	0.27	0.9	0.18
T7	1.3 (0.28)	1.43 (0.37)	0.2	0.33	0.87	0.23
log pNN50^a^						
T1	0.5 (0.64)	0.48 (0.8)	0.95	0.73	/	
T2	0.69 (0.58)	0.48 (0.62)	0.27	0.6	0.64	0.51
T3	0.42 (0.66)	0.23 (0.53)	0.31	0.6	0.73	0.63
T4	0.38 (0.58)	0.29 (0.48)	0.59	0.53	0.57	0.5
T5	0.34 (0.55)	0.09 (0.51)*	0.12	0.53	0.51	0.69
T6	0.48 (0.49)	0.55 (0.73)	0.73	0.62	0.58	0.55
T7	0.48 (0.42)	0.76 (0.71)*	0.13	0.58	0.52	0.49

*Note*: Values are means with standard deviation.

Abbreviations: DOC, disorders of consciousness; HRV, heart rate variability; pNN50, proportion of the number of times successive heartbeat intervals exceeded 50 ms divided by the total number of NN intervals, RMSSD: root mean square of successive differences; SDNN, standard deviation of normal‐to‐normal intervals; T1, 5‐min HRV data collection period (baseline supine position); T2 to T5, first to fourth 5‐min HRV data periods (75° tilted position); T6 to T7, first to second 5‐min HRV data collection period (supine position after tilt).

^a^ SDNN, RMSSD, and pNN50 were normalized by log_10_ transformation.

*p < 0.05, **p < 0.01, ***p < 0.001 compared with baseline.

Log SDNN showed a significant increase in both the control and DOC group during T6 (first 5‐min interval at 0° after posture change from 75° to supine, *p* < 0.05). Neither log RMSSD nor log pNN50 changed compared with T1 in either group. During T7 (second 5‐min interval at 0° after posture change from 75° to supine), Log pNN50 decreased significantly in controls only relative to T1 (*t* = −2.5, *p* = 0.02, Cohen's d = 0.49) (Figure 3a,c and Table 2).

Secondly, temporal changes of frequency‐domain outcomes, including log LF, log nLF, log HF, log nHF, and log LF/HF, were investigated for each group. No change was observed in log nLF or log HF during T2 compared to T1 in either group. Log nHF decreased and log LF increased in both the DOC and control group (*p* < 0.05), while log LF/HF was increased only in the control group relative to T1 (t = −3.2, *p* = 0.004, Cohen's d = 0.42). During T3–T5, log nLF and log LF/HF increased (*p* < 0.01) while log HF and log nHF decreased significantly in controls (*p* < 0.05). In DOC group, log LF/HF increased from T4 to T5, while log HF decreased at T5 and log nHF decreased at T3 and T5 compared to T1 (*p* < 0.05). No significant changes were observed in log LF compared with T1 for either group (Figure 3b,c and Table 3).

**TABLE 3:** HRV frequency‐domain outcomes in HUT test.

	DOC patients *n* = 21	Healthy controls *n* = 21	Inter‐group *p*‐Value	Intergroup effect size	Within‐group effect size	
					DOC	Healthy control
log LF^a^						
T1	1.93 (0.56)	2.4 (0.57)	0.01	0.57	/	
T2	2.24 (0.63)*	2.61 (0.42)**	0.03	0.54	0.68	0.31
T3	1.91 (0.68)	2.42 (0.54)	0.01	0.62	0.74	0.36
T4	1.88 (0.55)	2.48 (0.5)	0.001	0.53	0.69	0.37
T5	1.79 (0.59)	2.47 (0.5)	<0.001	0.55	0.69	0.43
T6	2.09 (0.59)	2.58 (0.46)*	0.005	0.53	0.67	0.36
T7	2.09 (0.62)	2.64 (0.65)*	0.007	0.63	0.52	0.44
log nLF^a^						
T1	1.28 (0.32)	1.49 (0.27)	0.03	0.3	/	
T2	1.16 (0.38)	1.46 (0.24)	0.004	0.32	0.35	0.35
T3	1.32 (0.35)	1.68 (0.16)**	<0.001	0.28	0.37	0.24
T4	1.45 (0.29)	1.69 (0.16)**	0.003	0.24	0.41	0.3
T5	1.34 (0.35)	1.76 (0.13)***	<0.001	0.26	0.46	0.26
T6	1.1 (0.41)*	1.38 (0.39)	0.03	0.4	0.39	0.43
T7	1.35 (0.37)	1.59 (0.2)	0.01	0.3	0.36	0.24
log HF^a^						
T1	2.09 (0.62)	2.45 (0.67)	0.08	0.65	/	
T2	2.3 (0.71)	2.37 (0.47)	0.7	0.6	0.65	0.58
T3	1.91 (0.89)	2.08 (0.52)**	0.46	0.73	0.76	0.47
T4	1.83 (0.79)	2.07 (0.51)**	0.25	0.66	0.65	0.53
T5	1.66 (0.86)*	1.96 (0.57)**	0.19	0.73	0.76	0.6
T6	1.99 (0.64)	2.32 (0.62)	0.09	0.63	0.7	0.38
T7	1.98 (0.66)	2.42 (0.87)	0.07	0.77	0.36	0.5
log nHF^a^						
T1	1.45 (0.33)	1.54 (0.27)	0.37	0.3	/	
T2	1.23 (0.38)*	1.22 (0.3)***	0.95	0.34	0.39	0.29
T3	1.32 (0.43)*	1.34 (0.22)**	0.83	0.34	0.29	0.28
T4	1.41 (0.25)	1.29 (0.19)***	0.09	0.22	0.24	0.27
T5	1.21 (0.39)**	1.25 (0.31)**	0.71	0.35	0.37	0.36
T6	1 (0.5)***	1.12 (0.36)***	0.37	0.44	0.45	0.32
T7	1.24 (0.34)**	1.38 (0.32)*	0.19	0.33	0.28	0.28
log LF/HF^a^						
T1	−0.17 (0.37)	−0.05 (0.47)	0.36	0.42	/	
T2	−0.06 (0.27)	0.24 (0.3)**	0.001	0.28	0.31	0.42
T3	−0.0003 (0.53)	0.34 (0.3)***	0.02	0.43	0.46	0.42
T4	0.05 (0.49)*	0.41 (0.31)***	0.007	0.41	0.38	0.48
T5	0.13 (0.45)**	0.51 (0.37)***	0.005	0.41	0.41	0.51
T6	0.1 (0.29)**	0.26 (0.46)**	0.19	0.38	0.37	0.45
T7	0.11 (0.43)**	0.22 (0.42)*	0.42	0.43	0.38	0.42

*Note*: Values are means with standard deviation.

Abbreviations: DOC, disorders of consciousness; HRV, heart rate variability; HF, high frequency; LF/HF, low to high‐frequency ratio; LF, low frequency; nHF, normalized high frequency; nLF, normalized low frequency; T1, 5‐min HRV data collection period (baseline supine position); T2 to T5, first to fourth 5‐min HRV data periods (75° tilted position); T6 to T7: first to second 5‐min HRV data collection period (supine position after tilt).

^a^ LF, nLF, HF, nHF, and LF/HF were normalized by log_10_ transformation.

*p < 0.05, **p < 0.01, ***p < 0.001 compared with baseline.

During T6, Log LF/HF increased significantly and nHF decreased in both DOC and control group compared with T1 (*p* < 0.01). Log LF increased (*t* = −2.31, *p* = 0.03, Cohen's d = 0.36) and log nLF was reduced (*t* = 2.19, *p* = 0.04, Cohen's d = 0.39) for control and DOC group compared with T1, respectively. During T7, no significant changes were observed in log nLF or log HF compared with T1 for either group. Log LF and log LF/HF increased (*t* = −2.87, *p* = 0.01, Cohen's d = 0.42) and nHF decreased (*t* = 2.62, *p* = 0.02, Cohen's d = 0.28) for controls relative to T1. In DOC group, log nHF was reduced (*t* = 3.49, *p* = 0.002, Cohen's d = 0.28) and log LF/HF was increasd (*t* = −3.32, *p* = 0.003, Cohen's d = 0.38) compared to T1. Log nLF and log HF did not change relative to T1 for either group (Figure 3b,c and Table 3).

### 3.4 Comparisons of HRV time‐ and frequency‐domain outcomes during HUT test between DOC patients and controls

Log LF and log nLF were significantly lower in the DOC group than in the control group from T1 to T7 (*p* < 0.05). Log LF/HF in the DOC group were significantly lower than in the control group during T2–T5 (*p* < 0.05) and log SDNN in the DOC group were significantly lower only during T4 (*t* = −2.54, *p* = 0.02, Cohen's d = 0.23). No significant between‐group differences were detected in log RMSSD, log pNN50, log HF, or log nHF from T1 to T7 (Tables 2 and 3).

## 4 DISCUSSION

Patterns of changes in HRV outcomes between DOC patients and healthy participants during HUT test and differences in ANS function between these two groups were explored. The results of two‐way measures ANOVA and *t*‐test suggest that these two groups shared a similar pattern of changes in HRV outcomes, as evidenced by gradually enhanced SNS (represented by log nLF) and diminished PNS (represented by log nHF) during the transition in body position from supine to tilted and enhanced PNS and diminished SNS when returning back to supine. Furthermore, there was a significantly delayed and attenuated autonomic response, especially in the SNS, in DOC patients throughout the HUT test, as evidenced by the delayed statistically significant increased in log nLF and log LF/HF compared with baseline and significant lower value of log LF, log nLF, and log LF/HF compared with healthy controls.

Decreased autonomic regulation may result from specific anatomical brain injuries. This association may be explained via the Central Autonomic Network (CAN) which comprises cortical regions of the brain, such as the medial prefrontal cortex, anterior cingulate cortex, and insular cortex; subcortical structures, such as the paraventricular nucleus, amygdala (central and lateral nuclei), and hypothalamic nuclei and midbrain regions (periaqueductal gray region) and pons (nucleus of the tractus solitarius, nucleus ambiguus, and ventrolateral medulla) (Benarroch, 1993). These structures modulate autonomic responses to various stimuli, including pain, emotions, behavior, and cognitive inputs (Riganello, Chatelle, Schnakers et al., 2019; Riganello, Larroque, Perri et al., 2019) and are associated with the regulation of consciousness (Edlow et al., 2021). Damage to these regions, resulting from CE, TBI, or HIE, may impair autonomic regulation and lead to a decline or loss of consciousness. Interactions between the central nervous system (CNS) and the ANS are likely to underly mechanisms behind autonomic dysfunction accompanying DOC.

HRV assessment during the HUT test represents a novel approach within the category of evoked tests. HRV assessments during musical and pain stimulation have been reported previously (Riganello et al., 2010, 2015; Riganello, Chatelle, Schnakers et al., 2019; Riganello, Larroque, Perri et al., 2019). The induction of HRV changes in real‐time by external non‐noxious or noxious stimuli during evoked assessments allows evaluation of ANS regulatory capacity in DOC patients in a more dynamic and efficient way. Music stimulation bears similarities to HUT as they can both serve as a non‐noxious evaluation tool combining with HRV for DOC patients' autonomic response and as means of arousal therapy. nLF and Sample Entropy (SampEn) were analyzed by Riganello et al. (2015) in 16 healthy individuals and nine VS patients before and after listening to four pieces of music with varying complexity and dynamic parameters. No significant differences in nLF or SampEn were seen between the two groups before listening to music but HRV outcomes in the DOC group were significantly attenuated as the complexity of the music increased compared to controls. Previous studies of music therapy have found meditative or calming music to be more for the psychological and cardiovascular systems of intensive care patients than metal or electronic music (Trappe, 2012). Indeed, soothing and structurally simple music was thought to activate the ANS and increase the chances of DOC patient arousal according to Riganello et al. Our study showed consistent significant changes to log LF/HF from the first 5‐min interval of 75° tilting (T1) to the second 5‐min interval of supine after tilting (T7) and log nLF from the second to the fourth 5‐min interval of 75° tilting (T3–T5) in healthy subjects compared to the baseline supine position (T1). By contrast, DOC patients did not show consistent significant changes to log LF/HF until T4 compared to T1 and log LF/HF in DOC patients were significantly lower than the healthy participants, suggesting a delayed and impaired sympathetic response in DOC patients during the HUT test. Moreover, HUT is a commonly applied arousal therapy to stimulate the activation of SNS for DOC patients with a degree of inclination between 60° and 90° and session duration of 10–20 min (Ng & King, 2021). The current findings suggest that the SNS of DOC patients may not be activated until at least 15 min after tilting to 75°, consistent with common HUT practice. However, the optimal duration of HUT has not been extensively studied, and assessment of optimal HUT duration for activation of autonomic responses with HRV as primary outcome may be valuable, given the functional and structural interactions between the ANS and CNS. Returning to HRV based on evoked tests, it is worth mentioning that HRV during HUT test appears to have some advantages over HRV during music stimulation. Firstly, up to 58% TBI patients have hearing impairment or loss, particularly when injuries are severe (Chen et al., 2018), limiting the use of music stimulation while not affecting the application of HUT test. Secondly, investigating HRV during the HUT test may also be more objective than that during musical stimulation which may be influenced by individual sensitivities to musical styles and melodies arising from variations in connectivity between subcortical reward system and higher‐order cortical areas (Martínez‐Molina et al., 2019). Thus, HRV during the HUT test encompasses a greater diversity of patients. However, further research is required to compare the diagnostic and differentiation efficacy of HRV evoked by these two tests for DOC patients.

We acknowledge some limitations to the current study, including a relatively small sample size which may limit statistical power. However, significant differences in multiple HRV outcomes were found between healthy participants and DOC patients, and future research with larger sample sizes may confirm the findings. Differences in HRV outcomes during the HUT test among DOC subtypes were not investigated due to small sample size and changes in blood pressure during the HUT test were not measured since the included DOC patients did not have cardiac dysfunction, nevertheless the inclusion of blood pressure changes would give a more comprehensive representation of autonomic response. Lastly, anatomical and functional changes of CNS were not investigated using neuroimaging techniques such as fMRI and near‐infrared spectroscopy and could not be correlated with HRV outcomes due to the large proportion (80.95%, 17/21) of DOC patients who had undergone cranioplasty (Table 1).

## 5 CONCLUSION

The change in values of HRV outcomes during the HUT test in DOC patients and healthy participants followed a similar pattern that SNS and PNS was gradually enhanced and diminished, respectively, during the transition in body position from supine to tilted, while PNS and SNS was gradually enhanced and diminished, respectively, when returning back to supine. However, compromised ANS regulatory function, particularly within the SNS, was observed in DOC patients. It is essential to note that log nLF and log LF/HF in DOC patients changed in a delayed manner and log LF, log nLF, and log LF/HF were significantly lower throughout the titled phase compared with healthy participants.

The current study contributes to the limited body of research regarding ANS function during the HUT test in DOC patients. The importance of assessing autonomic dysfunction as an integral component of DOC clinical evaluations is stressed. HRV assessment during the HUT test may potentially offer a more efficient means of gaining insights into DOC patients' neurological functions and prognoses.

## AUTHOR CONTRIBUTIONS

Weiqiang Cai and Xu Han conducted data acquisition, analysis, and manuscript drafting. Hongyu Xie and Yi Wu designed and supervised the work. Xinwei Tang, Zuojun Cao, Zi Yu, Zuowen Sun, and Junfa Wu contributed to design, data interpretation, and manuscript revision. All authors reviewed and approved the manuscript, jointly assuming responsibility for the work.

## FUNDING INFORMATION

This study was supported by the National Key R&D Program of China (No. 2022YFC3601204), National Key R&D Program of China (No. 2018YFC2001700), Key Supporting Discipline Construction Project of Shanghai Health System (No. 2023ZDFC0304), Shanghai Science and Technology Innovation Action Plan (No. 20412420200), Shanghai Municipal Key Clinical Specialty (No. shslczdzk02702), and the Scientific Research Program of Huashan Hospital (No. 2021QD031).

## CONFLICT OF INTEREST STATEMENT

The authors declare that they have no conflict of interest. The funders had no role in the design, analyses, interpretation of data, writing of the manuscript, or in the decision to publish the results.

## ETHICS STATEMENT

Ethical approval was granted by the Ethics Committee of Huashan Hospital (2023 ‐ 469).

## INFORMED CONSENT

Written informed consent was obtained from all healthy participants and patients' legal representatives.

## TRIAL REGISTRATION

This study protocol was registered at Chinese Clinical Trial Registry (ChiCTR230007371).

## Supporting information


Table S1.

Table S2.

## Data Availability

The datasets used and analyzed during the current study are available from the corresponding authors on reasonable request.
