# Supplementary material for: Uncovering sympathetic nervous system dysfunction in disorders of consciousness via heart rate variability during head‐up tilt test
Source: Physiol Rep. 2024 Apr 7;12(7):e16000. doi: 10.14814/phy2.16000 (PMC10999365; doi:10.14814/phy2.16000)
Supplement: Supplementary file 1 — Table S1. [file PHY2-12-e16000-s001.docx]

**SUPPLEMENTARY TABLE 1** Two-way repeated measures ANOVA results of HRV time-domain outcomes. HRV: heart rate variability; SS: sum of squares; Df: degree of freedom; MS: mean squares; η_p_^2^: partial eta-squared; SDNN: standard deviation of normal-to-normal intervals; RMSSD: root mean square of successive differences; pNN50: proportion of the number of times successive heartbeat intervals exceeded 50ms divided by the total number of NN intervals. ^†^SDNN, RMSSD, and pNN50 were normalized by log_10_ transformation.

| Source of varation | SS | Df | MS | F | *p*-value | η_p_^2^ |
| --- | --- | --- | --- | --- | --- | --- |
| log SDNN^†^ | | | | | | |
| Between-subject | | | | | | |
| Group | 0.8 | 1 | 0.8 | 3.1 | 0.09 | 0.072 |
| Error (group) | 10.37 | 40 | 0.26 |  |  |  |
| Within-subject | | | | | | |
| Position | 2.83 | 4.36 | 0.65 | 16.05 | <0.001 | 0.29 |
| Interaction | 0.12 | 4.36 | 0.03 | 0.69 | 0.62 | 0.02 |
| Error (position) | 7.05 | 203.25 | 0.04 |  |  |  |
| log RMSSD^†^ | | | | | | |
| Between-subject | | | | | | |
| Group | 0.13 | 1 | 0.13 | 0.31 | 0.58 | 0.008 |
| Error (group) | 16.95 | 39 | 0.44 |  |  |  |
| Within-subject | | | | | | |
| Position | 2.25 | 4.04 | 0.56 | 10.83 | <0.001 | 0.22 |
| Interaction | 0.23 | 4.04 | 0.06 | 1.09 | 0.37 | 0.03 |
| Error (position) | 8.1 | 157.5 | 0.05 |  |  |  |
| log pNN50^†^ | | | | | | |
| Between-subject | | | | | | |
| Group | 0.26 | 1 | 0.26 | 0.15 | 0.7 | 0.004 |
| Error (group) | 69.18 | 40 | 1.73 |  |  |  |
| Within-subject | | | | | | |
| Position | 5.68 | 4.1 | 1.39 | 7.01 | <0.001 | 0.15 |
| Interaction | 2.23 | 4.1 | 0.54 | 2.75 | 0.03 | 0.06 |
| Error (position) | 32.4 | 163.96 | 0.2 |  |  |  |
