# Supplementary material for: Uncovering sympathetic nervous system dysfunction in disorders of consciousness via heart rate variability during head‐up tilt test
Source: Physiol Rep. 2024 Apr 7;12(7):e16000. doi: 10.14814/phy2.16000 (PMC10999365; doi:10.14814/phy2.16000)
Supplement: Supplementary file 2 — Table S2. [file PHY2-12-e16000-s002.docx]

**SUPPLEMENTARY TABLE 2** Two-way repeated measures ANOVA results of HRV frequency-domain outcomes. HRV: heart rate variability; SS: sum of squares; Df: degree of freedom; MS: mean squares; η_p_^2^: partial eta-squared; LF: low frequency; nLF: normalized low frequency; HF: high frequency; nHF: normalized high frequency; LF/HF: low to high-frequency ratio. ^†^LF, nLF, HF, nHF, and LF/HF were normalized by log_10_ transformation.

| Source of varation | SS | Df | MS | F | *p*-value | η_p_^2^ |
| --- | --- | --- | --- | --- | --- | --- |
| log LF^†^ | | | | | | |
| Between-subject | | | | | | |
| Group | 20.34 | 1 | 20.34 | 13.49 | <0.001 | 0.25 |
| Error (group) | 60.29 | 40 | 1.51 |  |  |  |
| Within-subject | | | | | | |
| Position | 3.56 | 4.79 | 0.74 | 4.79 | <0.001 | 0.11 |
| Interaction | 29.73 | 4.79 | 0.13 | 0.86 | 0.51 | 0.02 |
| Error (position) | 29.73 | 165.44 | 0.18 |  |  |  |
| log nLF^†^ | | | | | | |
| Between-subject | | | | | | |
| Group | 6.33 | 1 | 6.33 | 25.43 | <0.001 | 0.39 |
| Error (group) | 9.96 | 40 | 0.25 |  |  |  |
| Within-subject | | | | | | |
| Position | 4.02 | 4.39 | 0.92 | 10.29 | <0.001 | 0.21 |
| Interaction | 0.37 | 4.39 | 0.08 | 0.94 | 0.45 | 0.02 |
| Error (position) | 15.62 | 175.54 | 0.09 |  |  |  |
| log HF^†^ | | | | | | |
| Between-subject | | | | | | |
| Group | 5.5 | 1 | 5.5 | 2.48 | 0.12 | 0.06 |
| Error (group) | 88.84 | 40 | 2.22 |  |  |  |
| Within-subject | | | | | | |
| Position | 8.95 | 4.18 | 2.14 | 8.49 | <0.001 | 0.18 |
| Interaction | 0.98 | 4.18 | 0.23 | 0.93 | 0.45 | 0.02 |
| Error (position) | 42.19 | 167.17 | 0.25 |  |  |  |
| log nHF^†^ | | | | | | |
| Between-subject | | | | | | |
| Group | 0.12 | 1 | 0.12 | 0.29 | 0.59 | 0.007 |
| Error (group) | 24.55 | 40 | 0.61 |  |  |  |
| Within-subject | | | | | | |
| Position | 4.56 | 4.17 | 1.09 | 11.64 | <0.001 | 0.23 |
| Interaction | 0.49 | 4.17 | 0.12 | 1.24 | 0.3 | 0.03 |
| Error (position) | 15.69 | 166.85 | 0.09 |  |  |  |
| log LF/HF^†^ | | | | | | |
| Between-subject | | | | | | |
| Group | 4.72 | 1 | 4.72 | 7.69 | 0.008 | 0.16 |
| Error (group) | 24.55 | 40 | 0.61 |  |  |  |
| Within-subject | | | | | | |
| Position | 4.53 | 4.13 | 1.1 | 9.14 | <0.001 | 0.19 |
| Interaction | 0.91 | 4.12 | 0.22 | 1.84 | 0.12 | 0.04 |
| Error (position) | 19.82 | 164.99 | 0.12 |  |  |  |
